# Effects of *DNMT1* and *HDAC* Inhibitors on Gene-Specific Methylation Reprogramming during Porcine Somatic Cell Nuclear Transfer

**DOI:** 10.1371/journal.pone.0064705

**Published:** 2013-05-31

**Authors:** Weihua Xu, Zicong Li, Bo Yu, Xiaoyan He, Junsong Shi, Rong Zhou, Dewu Liu, Zhenfang Wu

**Affiliations:** 1 Department of Animal Genetics, Breeding and Reproduction, South China Agricultural University, Guangzhou, Guangdong, China; 2 College of Life Science, Longyan University, Longyan, Fujian, China; 3 Wen’s Research Institute, Yunfu, Guangdong, China; Institute of Zoology, Chinese Academy of Sciences, China

## Abstract

Somatic cell nuclear transfer (SCNT) in mammalian cloning currently remains inefficient. Incomplete or erroneous epigenetic reprogramming of specialized donor somatic nuclear and resulting aberrant gene expression during development of cloned embryos is commonly believed as the main reason that causes the low efficiency of SCNT. Use of small molecular reprogramming modifiers to assist the somatic nucleus to mimic naturally occurring DNA methylation and chromatin remodeling in nucleus of fertilization-derived zygotes, has been widely attempted to improve cloning efficiency. However, impacts of these small modifiers on gene-specific methylation dynamics and their potential effects on methylation of imprinted gene have rarely been traced. Here, we attempted two relatively novel *DNMT1* inhibitor (DNMTi) and histone deacetylase inhibitor (HDACi), scriptaid and RG108, and demonstrated their effects on dynamics of gene-specific DNA methylation and transcription of porcine SCNT embryos. We found that scriptaid and RG108 had synergetic effects on rescuing the disrupted methylation imprint of *H19* during SCNT at least partially by repression over-expressed *MBD3* in eight-cell cloned embryos. Furthermore, we firstly identified a differential methylation regions (DMRs) at 5′ flanking regions of *XIST* gene and found that scriptaid alone and its combination with RG108 modify the dynamics of both transcription and DNA methylation levels in cloned embryos, by different manners. Additionally, we found that scriptaid alone and its combination with RG108 can significantly promote the transcription of *NANOG* in cloned embryos and enhance their pre-implantation developmental capacity. Our results would contribute to uncovering the epigenetic reprogramming mechanisms underlying the effects of assisted small molecules on improvement of mammalian cloning efficiency.

## Introduction

So far, applications of cloned pigs in biomedicine and agriculture by somatic cell nuclear transfers (SCNT) have been achieved greatly [1]. Despite these achievements, SCNT technology in pigs, as it does in most mammals, remains inefficient and cloning efficiency is usually around 1–5% of embryos transferred surviving to term [2]–[4]. The prevailing view is that incomplete epigenetic reprogramming of donor cell nuclei and resulting aberrant gene expression during development [2], [5]–[7]. To facilitate nuclear reprogramming and thus improve cloning efficiency, several methods, including treating the donor cells and/or early nuclear transferred embryos with DNMT1 inhibitors (DNMTi) like 5-aza-20-deoxycytidine (5-aza-dC) and histone deacetylatse inhibitors (HDACi) like TSA and scriptaid, have been tested to assist the somatic nucleus to mimic DNA methylation and chromatin remodeling[6], [8]–[9].

Scriptaid, one of HDACi, conferred the greatest effect and with low toxicity that enhances transcriptional activity and protein expression [10], has especially been focused in recent years and found beneficial in improving cloning successful rate and correcting gene expression in pigs [4], [11]. RG108, a novel DNMT1 inhibitor, was tested solely free of cytotoxic or genotoxic effects compared to the other five DNMT1 inhibitors (5-aza-CR, 5-aza-CdR, zebularine, procaine and epigallocatechin-3-gallate) in human cell lines [12], [13]. In mouse, cloned embryos treated with 500 µM RG108 from the two-cell to morula/blastocyst stage, higher *POU5F1* expression and more ICM cells were observed [14].

To our knowledge, rare reports have been reported on combined use of scriptaid and RG108 in porcine SCNT. We attempted to treat porcine nuclear transfer embryos after fusion for 17∼19 hours with RG108 alone, scriptaid alone and their combination, and observed positive effects of scriptaid alone or along with RG108 on in vitro developmental capacity during pre-implantation except with RG108 alone, unexpectedly, we found their combination could rescue the disrupted methylation imprints at *H19* locus and significantly reduced RNA levels of *XIST* in male cloned blastocysts. A preceded report [15] and our study both observed unfaithful maintenance of methylation imprint at *H19* locus during SCNT. Moreover, Inhibition of *XIST* in cloned embryos may be vital because a research group consecutively reported *XIST* was aberrantly transcribed in cloned mice and bovine early embryos and depletion or inhibition of *XIST* gene dramatically improved cloning efficiency in mice [16], [17]. Thereafter, we focused on *H19* and *XIST* genes and traced the potential impacts on methylation dynamics of *H19* and *XIST* genes during pre-implantation by scriptaid alone and its combination with RG108.

## Results

### Scriptaid Alone and its Combination with RG108 can Improve Developmental Capacity of Cloned Embryos

To determine the optimum addition of RG108, We firstly designed three levels of RG108 (100 µM, 200 µM, 400 µM) to observe potential cytotoxicity to donor adult fibroblasts and found 400 µM RG108 displayed an obvious deleterious effect on cell proliferation (Figure S1 in File S1). We then compared their effects on developmental potentials and obtained the highest average total cells of blastocysts at the moderate levels of RG108 (200 µM) (P<0.05, Table S1 in File S2). 500 nM scriptaid was used in previous reports [4], [11]; herein we set a lower level of scriptaid (100 nM) and compared its effect on embryos developmental capacity with the reported levels (500 nM). We found 100 nM achieved similarly in the aspects of blastocyst rate and average total cell number of blastocysts (P>0.05, Table S2 in File S2). Therefore, we determined the optimum levels of RG108 (200 µM) and scriptaid (100 nM).

We found scriptaid alone and its combination with RG108 significantly improved the blastocysts ratio and the total cells of blastocysts, whereas RG108 alone did not (Table 1). No significant difference was found between Scr-NT group (24.60% of % blast and 50.00±1.45 of average total cells) and RG+Scr-NT group (29.31% of % blast and 50.00±2.00 of average total cells) (Table 1). To explore the potential impacts at the molecular levels, we evaluated the possible alterations on genes transcription.

**Table 1:** The effects of RG108, Scriptaid and RG108 plus scriptaid on development of porcine SCNT embryos in vitro¶.

Treatment	% cleaved (n)	% blast (n)	Average total cells±SEM
**Con-NT**	82.42(211/256)	19.14(49/256)^a^	37.33±1.45^a^
**RG-NT**	81.79(247/302)	18.21(55/302)^a^	42.33±1.45^ab^
**Scr-NT**	79.76(201/252)	24.60(62/252)^b^	50.00±2.00^b^
**RG+Scr-NT**	80.75(281/348)	29.31(102/348)^b^	52.00±3.94^b^

¶ (1) the proportion of two-cell embryos (% cleaved) and the proportion of blastocysts (% blast) from 3 replicates; (2) n, number of embryos; (3) ratio or means labeled with the same letter or not labeled any letter do not differ from each other (p>0.05) and without the same letter differ significantly (p<0.05).

### Transcription Alterations of Twelve Selected Genes in Treated Cloned Blastocysts

**Figure 1:**
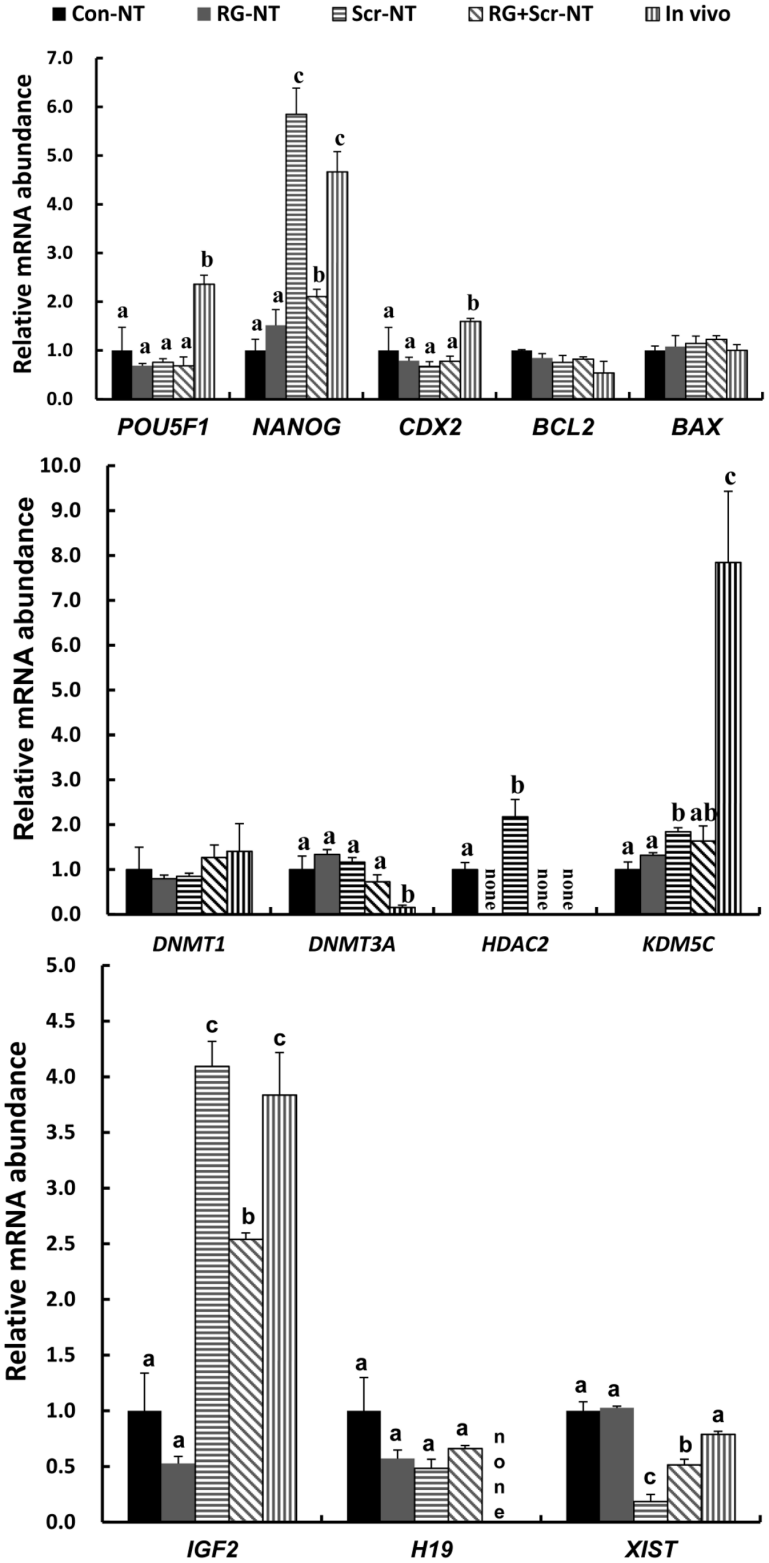
Comparison of transcription levels of twelve selected genes in blastocysts after treatment by RG108 and/or scriptaid. Con-NT, RG-NT, Scr-NT and RG+Scr-NT indicated groups untreated, RG108 alone, scriptaid alone or by RG108 and scriptaid simultaneously, respectively. In vivo produced blastocysts (in vivo) served as the reference. Bars replaced by the “none” indicated no detected transcription. Letters of “a, b, c” on bars referred to significant (P<0.05) differences. Means labeled with the same letter or no letters didn’t differ from each other (p>0.05) and without the same letter differed significantly (p<0.05).

We empirically selected twelve genes which consisted of pluripotency- (*POU5F1*, *NANOG*), apoptosis- (*BCL2*, *BAX*), lineage differentiation- (*CDX2*), DNA methylation- (*DNMT1*, *DNMT3A*), chromatin modification- (*HDAC2*, *KDM5C*), imprinted- (*IGF2*, *H19*) and X chromosome inactivation-related (*XIST*) genes in 7-day blastocysts derived from treated embryos with in vivo produced blastocysts (in vivo) as the reference. Compared with in vivo blastocysts, cloned embryos(Con-NT) displayed down-regulated expressions of genes including *POU5F1*, *NANOG*, *CDX2*, *IGF2* and *KDM5C* (P<0.05), whereas up-regulated expression of *HDAC2* and *H19* which were not detected in in vivo produced blastocysts. In the cases of treated embryos, addition of RG108 (RG-NT) completely repressed the expression of *HDAC2* (P<0.05) while had no significant effects on the other genes (P>0.05) (Figure 1). In Scr-NT blastocysts, *NANOG*, *HDAC2*, *KDM5C* and *IGF2* were significantly up-regulated, while *XIST* was down-regulated (P<0.05, Figure 1). In RG+Scr-NT blastocysts, *NANOG* and *IGF2* were up-regulated, whereas *XIST* and *HDAC2* were down-regulated (P<0.05, Figure 1). No differences were found between Scr-NT group and RG+Scr-NT group except for *HDAC2*, because *NANOG*, *KDM5C* and *XIST* displayed the same shift either up or down, though they differed in degrees.

**Figure 2:**
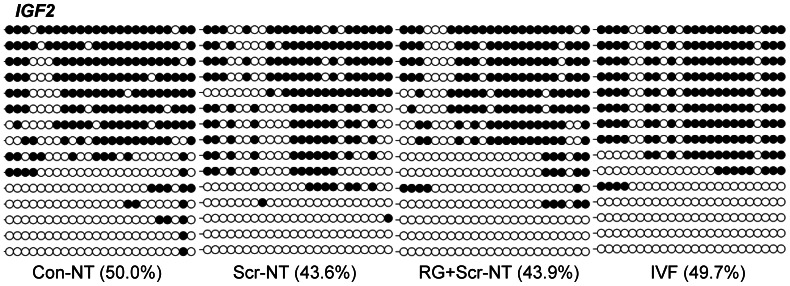
The effects RG108 and/or scriptaid on DNA methylation levels of *IGF2* DMR2 at blastocyst stage. No obvious DNA methylation alteration in treated embryos was found after treatment by RG108 and/or scriptaid on DNA methylation.

*IGF2* and *XIST* are commonly believed conformed to epigenetic modifications. Herein, we didn’t found obvious DNA methylation alteration at the DMR2 (a previously reported imprinted differentially methylated region in [18]) of *IGF2* among Con-NT embryos (50.0%), Scr-NT embryos (43.6%), RG+scr-NT embryos (43.9%) and IVF counterparts (49.7%) at the blastocyst stage (Figure 2). Imprints of *H19* and its role in controlling *IGF2* expression are illustrated as a model of epigenetic regulation of genes [19], and unfaithful maintenance of methylation imprint at *H19* locus during SCNT has been observed [15].In addition, it has been reported that depletion or repression of *XIST* expression dramatically improved cloning efficiency in mice [16], [17].Therefore, we determined to investigate the potential alterations at the levels of methylation of *H19* and *XIST* by HDACi and/or DNMTi during early embryonic development.

### RG108 and Scriptaid Rescued the Disrupted Methylation Imprinting of *H19* Gene by SCNT

**Figure 3:**
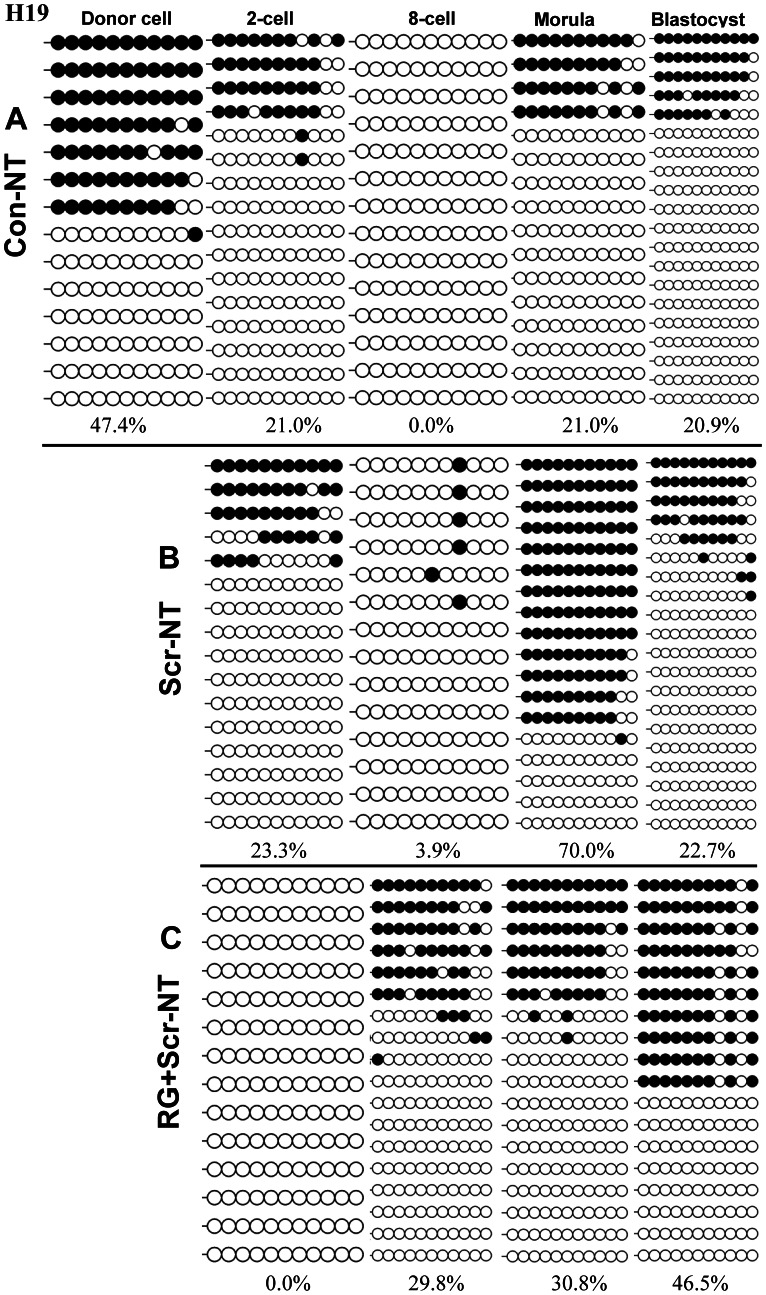
The effects of RG108 and/or scriptaid on the dynamics of DNA methylation at ICR3 of *H19* during SCNT. RG108 and scriptaid demonstrated synergetic effects on shortening the demethylation window around one division cycle with complete de-methylation at two-cell stage and re-methylation at eight-cell stage compared with the DNA methylation dynamics of Con-NT and Scr-NT embryos. At the blastocyst stage, embryos treated by RG108 and scriptaid almost rescued the semi-methylated status at *H19* ICR3 compared with IVF counterparts (Figure 4 A) while not for the other two groups.

We didn’t detected transcription of *H19* until at the morula stage in all cloned embryos and in vitro fertilized embryos, therefore, we solely investigated and compared the methylation dynamics of imprinting control region 3(ICR3) of *H19*, a well characterized differentially methylated region [18], by analyzing DNA methylation status in embryos at four developmental stages (two-cell, eight-cell, morulas and blastocysts). Imprinted genes are commonly believed capable of escaping DNA methylation reprogramming in early embryonic development [20], [21]. However, we observed the process of de-methylation and re-methylation in all cloned embryos, though they differed temporally (Figure 3). In Con-NT embryos, *H19* was fully demethylated at eight-cell stage and partially restored at the morula stage (Figure 3 A), whereas in RG+Scr-NT embryos, de-methylation was shifted earlier at two-cell stage and restored at eight-cell stage (Figure 3 C). For embryos treated with scriptaid alone, the process of demethylation was similar to untreated embryos, whereas an over-established methylation process at the morula stage and a subsequent drop of methylation at the blastocysts stage occurred (Figure 3 B).

**Figure 4:**
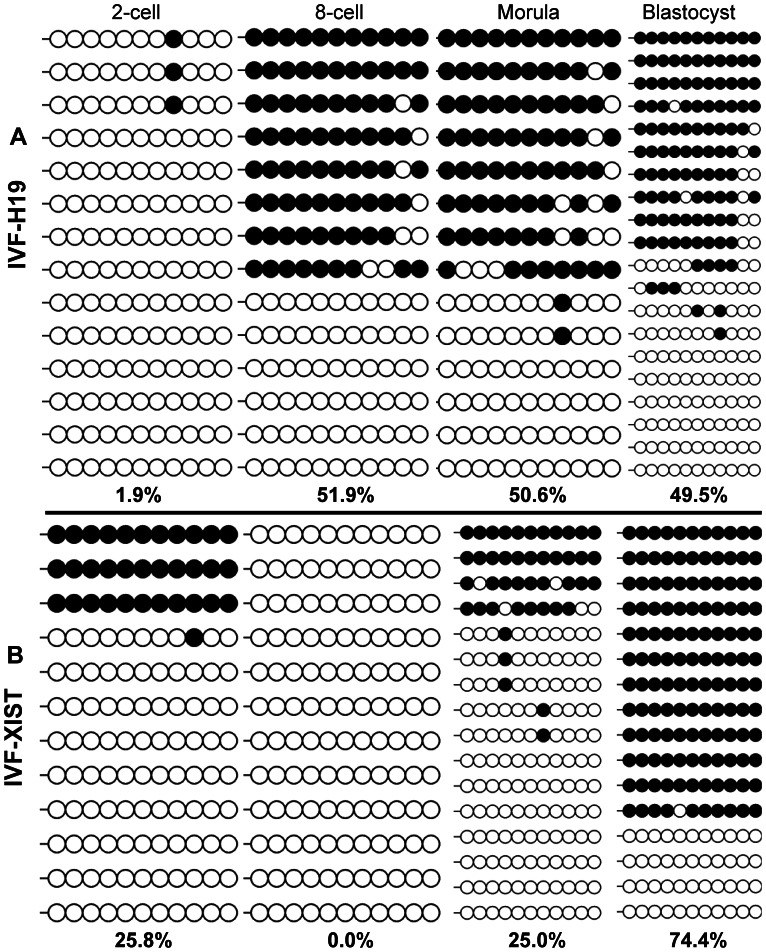
DNA methylation dynamics of *XIST* and *H19* genes in early IVF embryos. All embryos were sex-mixed except for those at morula and blastocyst stages for *XIST* gene which were male.

Notably, at the blastocyst stage, embryos treated by RG108 and scriptaid (RG+scr NT) almost rescued the imprinted and semi-methylated status at ICR3 of *H19* to the levels in IVF counterparts (Figure 4 A) while not for the Con-NT and scr-NT embryos (Figure 3 A, B). These results strongly suggested that HDACi and DNMTi had synergetic effects on maintaining faithful DNA methylation reprogramming of ICR3 of *H19*.

### Potential Links between the Rescued Imprinted Methylation at ICR3 of *H19* by RG108 and Scriptaid with the Expression Levels of *MBD3* in Eight-cell Stage Cloned Embryos

**Figure 5:**
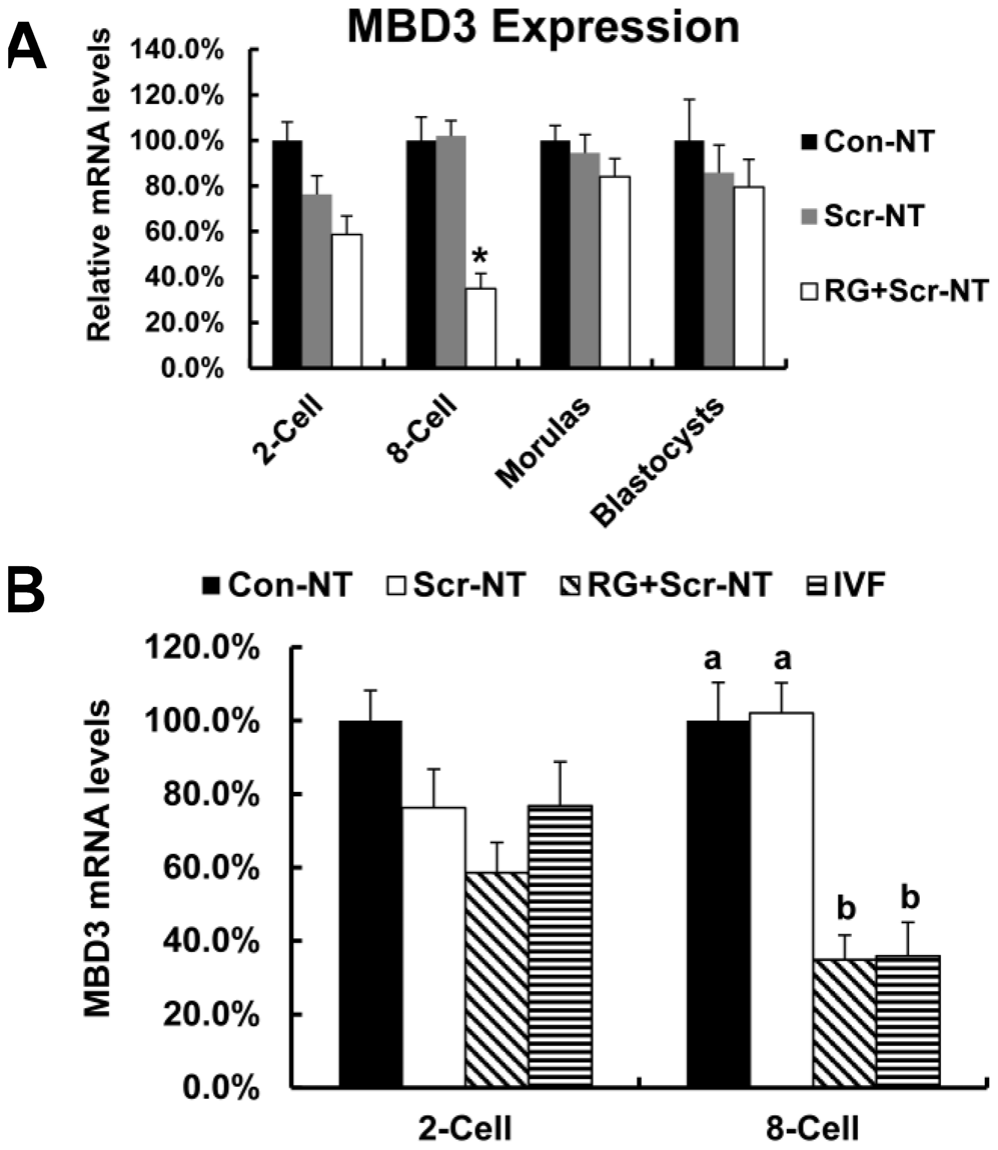
*MBD3* expression was affected by RG108 and scriptaid at eight cell stage. Treatment with RG108 and/or scriptaid can correct *MBD3* levels at 8-cell stage (A) which are overexpressed in Con-NT and Scr-NT embryos compared with IVF counterparts (B).

To explore the mechanisms underlying the rescued DNA methylation at ICR3 of *H19* by RG108 and scriptaid, we checked the expressions of *DNMT1* and *DNMT3A* during embryonic development but found no significant differences among cloned embryos and IVF embryos (data not shown). However, we detected a significant decreased *MBD3* mRNA levels in RG+Scr-NT embryos at eight-cell stage (P<0.05, Figure 5 A) which was comparable to that of in vitro fertilized counterparts (Figure 5 B).

**Figure 6:**
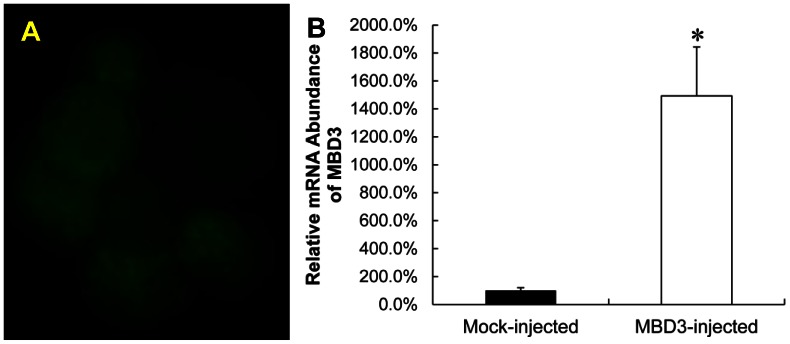
Overexpression of *MBD3* at eight cell stage. To exclude the embryos without over-expression due to failure of injection or import of plasmid DNA into the nucleus, the same molar ratio of CMV promoter-driven *MBD3*-coding plasmid and eGFP-coding plasmid were co-injected into the cytoplasmic of constructed oocytes, only those embryos with green fluorescence were picked(A).In addition, over-expressed *MBD3* was also validated by quantitative PCR (B).

We then constructed a CMV promoter-driven *MBD3*-coding plasmid and injected 10 pL plasmid solution (10 ng/µL plasmid) into the cytoplasmic of constructed oocytes with the culture medium supplemented with RG108 and scriptaid. To exclude the embryos without over-expression due to failure of injection or import of plasmid DNA into the nucleus, the same molar ratio of CMV promoter-driven eGFP-coding plasmid were co-injected, and only those embryos with green fluorescence were picked (Figure 6 A).In addition, over-expressed *MBD3* was also validated by quantitative PCR (Figure 6 B).

**Figure 7:**
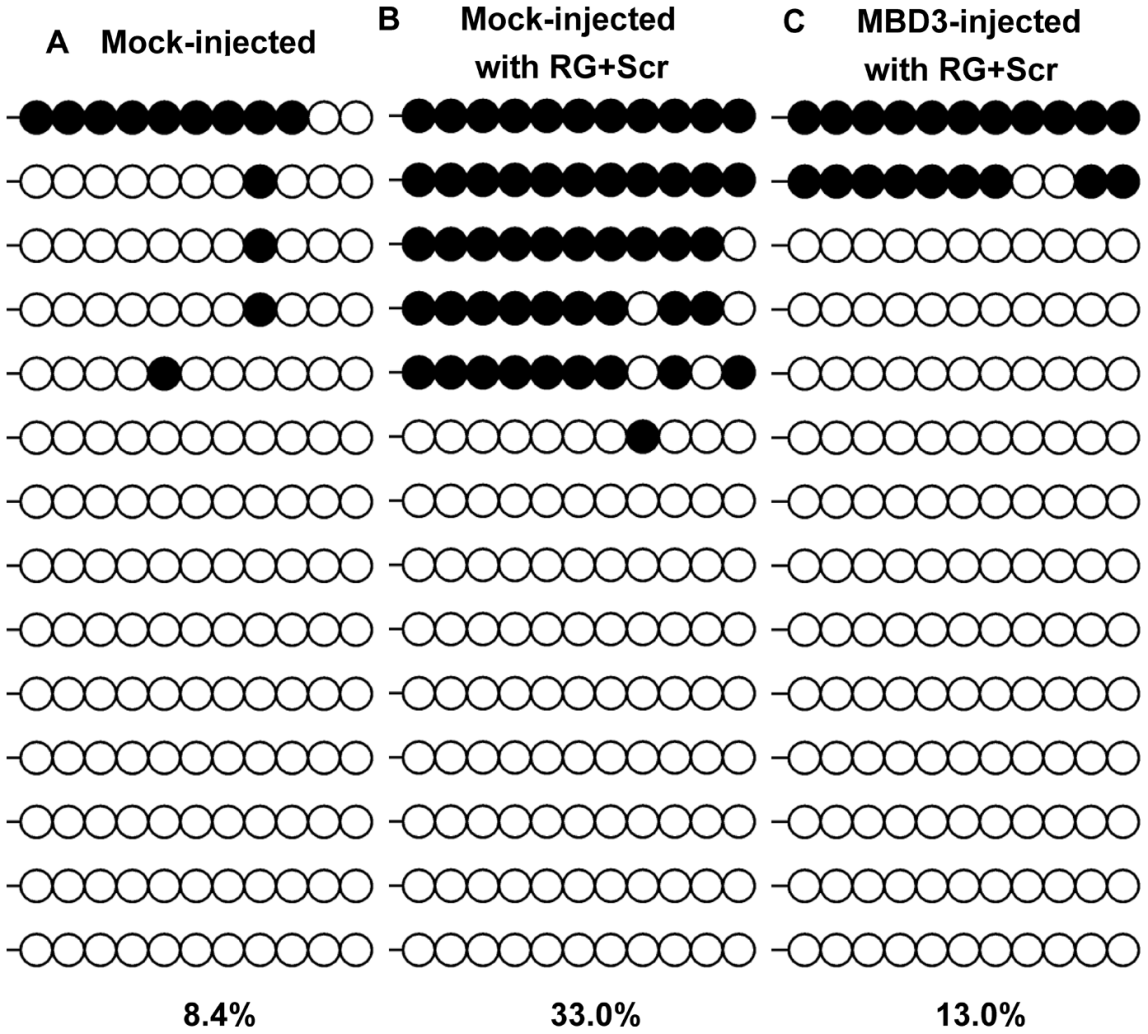
Effect of over-expression of *MBD3* at eight cell stage on DNA methylation levels when addition of RG108 and scriptaid. Co-injection with eGFP-coding plasmid and pcDNA3.1+ empty plasmid with equal molar ratio (A); mock injection with the addition of RG108 and scriptaid in the culture medium (B); *MBD3*-injected with RG+scr (C), co-injection with *MBD3*-coding plasmid and eGFP-coding plasmid with medium supplemented with RG108 and scriptaid.

We compared the DNA methylation levels at ICR3 region of *H19* among embryos at eight cell stage, and found the raised DNA methylation level by RG108 and scriptaid (33.0%, Figure 7 B) compared with that in mock injected embryos (8.4%, Figure 7 A), could be reduced dramatically (13.0%, Figure 7 C) due to the overexpression of *MBD3*. Our result might suggest that RG108 and scriptaid rescued the imprinted DNA methylation status at ICR3 of *H19* at least partially by repressing over-expressed *MBD3* in cloned embryos at eight-cell stage.

### Identification of Differential Methylation Regions (DMRs) of Porcine *XIST* Gene 5′ Flanking Regions

**Figure 8:**
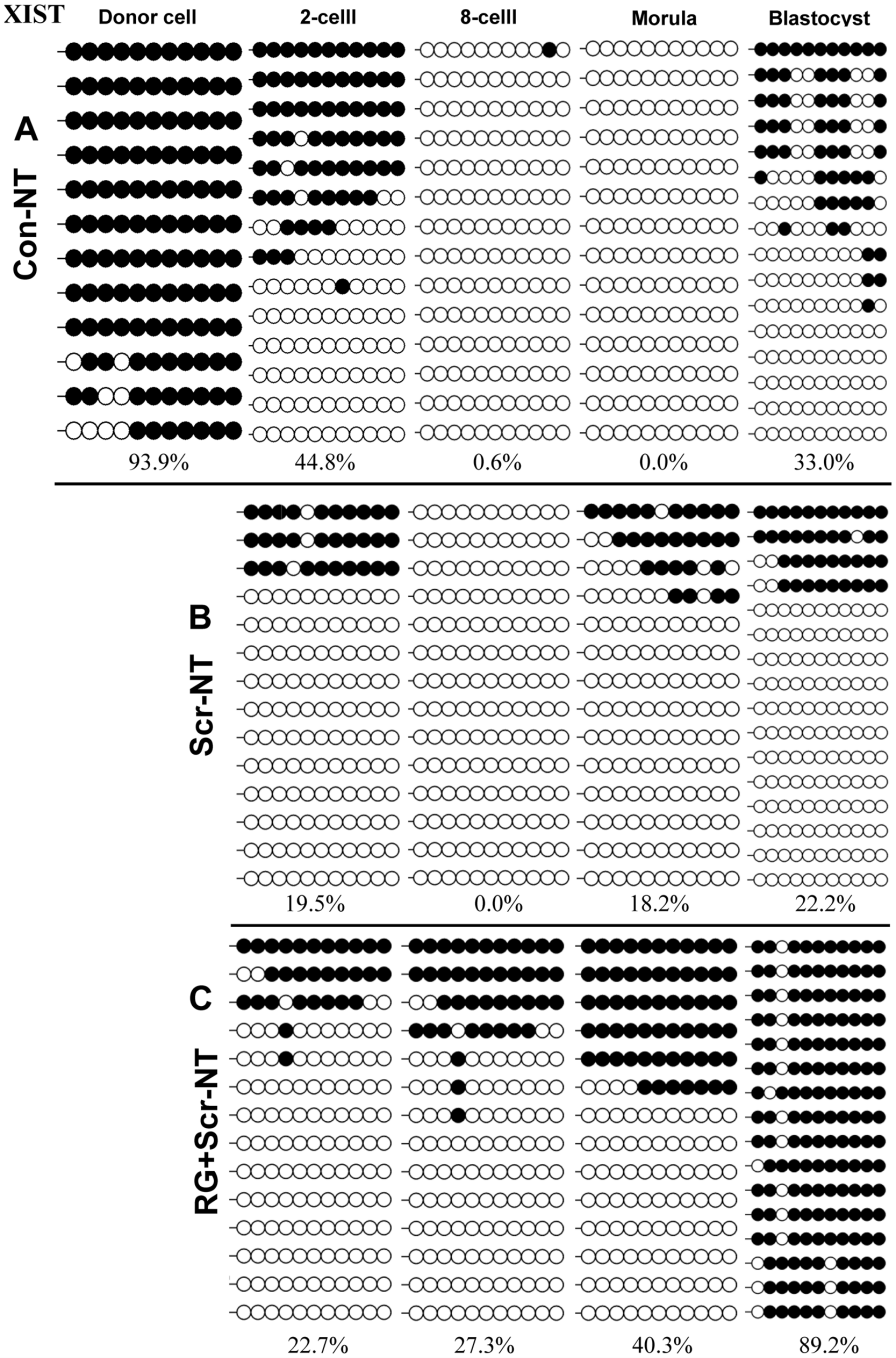
The effects of RG108 and/or scriptaid on the dynamics of DNA methylation reprogramming of *XIST* during SCNT. The 5′ upstream region of *XIST* gene in Con-NT embryos undertook de-methylation until at the morula stage (A), whereas a narrowed window of de-methylation occurred in both treated embryos (B, C).In addition, embryos treated by RG108 and scriptaid presented a partial de-methylation and almost fully established DNA methylation (89.2%) at blastocysts stage, which was even closer to the levels of IVF blastocysts (74.4%, Figure 4 B) compared with that of the Con-NT (33.0%) and Scr-NT (22.2%) embryos.

We blasted X chromosome along the sequence of EF619477.1 (Figure S2 A in File S1) and found an area containing two typical CpG islands (Figure S2 B,C in File S1) which were highly similar to the promoter and exon1 of bovine (NR_001464.2) and horse (U50911.1) respectively. Two CpG islands were differentially methylated in male and female genome of porcine adult fibroblasts and therefore defined as DMRs (Figure S2 F in File S1). We focused on DMR2 because it solely presented a dynamic methylation pattern during SCNT (Figure 8 A).

### The Effects of RG108 and Scriptaid on the Dynamics of DNA Methylation and Transcription of *XIST* during SCNT

**Figure 9:**
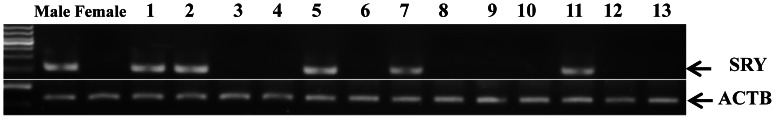
Sexing of IVF embryos. Porcine *SRY* gene specific nested primers were designed to distinguish the sex of IVF embryos.

Considering that X chromosome dosage compensation occurred in porcine embryos at the blastocyst stage [22], we made IVF embryo sexing at the morula and blastocyst stages (Figure 9).

Firstly, we traced the DNA methylation dynamics at DMR2 of *XIST* among embryos. We found untreated cloned embryos (Con-NT) extended de-methylation process across three division cycles from two-cell stage until the morula stage (Figure 8 A), whereas treated embryos (Scr-NT and RG+Scr-NT ) re-methylated at morula stage and presented a narrowed de-methylation window with two division cycles (Figure 8 B,C ). Additionally, embryos treated by RG108 and scriptaid almost fully established DNA methylation (89.2%, Figure 8 C) at the blastocyst stage, which was even closer to the levels of IVF blastocysts (74.4%, Figure 4 B) compared with that of the Con-NT (33.0%, Figure 8 A) and Scr-NT embryos (22.2%, Figure 8 B).

**Figure 10:**
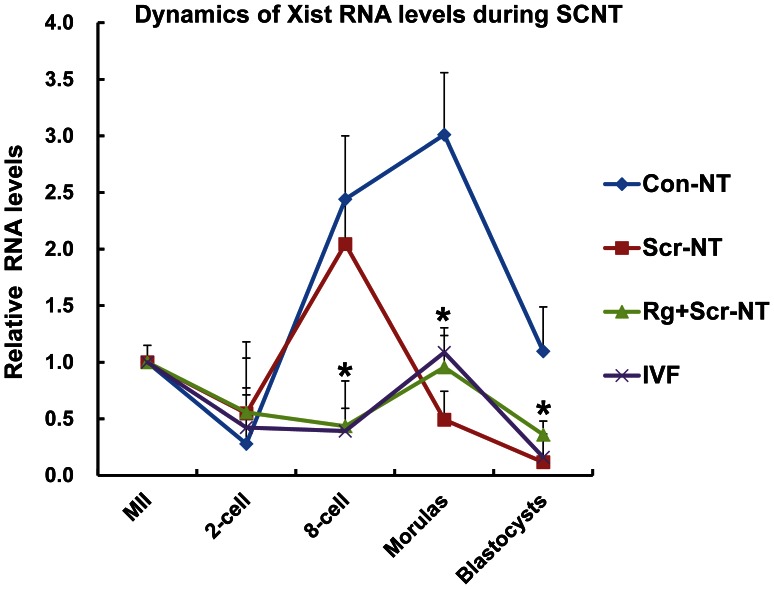
The effects of RG108 and/or scriptaid on the transcription dynamics of *XIST* gene. RG+Scr-NT embryos displayed a narrowed and moderate transcription activation and inactivation window similar to IVF counterparts, compared with the patterns of Con-NT and Scr-NT embryos. All embryos were sex-mixed except for those at the morula and blastocyst stages.

We also investigated the dynamics of transcription by quantitative PCR and found *XIST* was fully re-activated at the morula-stage in Con-NT and RG+Scr-NT embryos, whereas shifted earlier at eight-cell stage in Scr-NT embryos (Figure 10), furthermore, embryos treated with RG108 and scriptaid (RG+Scr-NT) exhibited a similar narrowed reactivation window and a minor expression peak as IVF counterparts (Figure 10), which fit well with a partial de-methylation and fully established methylation in RG+Scr-NT embryos (Figure 8 C).

## Discussion

Inhibiting HDACs with TSA was reported to result in pluripotent gene expressions [23], [24]. We herein found scriptaid alone and its combination with RG108 significantly improved expression of *NANOG* but not for *POU5F1* which was same to TSA treatment [25]. *NANOG* is believed the gateway to the pluripotent ground state, without *NANOG*, pluripotency does not develop, and the inner cell mass is trapped in a pre-pluripotent, indeterminate state [26]. From this aspect, Scriptaid alone and its combination of RG108 might facilitate reprogramming of cloned embryos to a more matured pluripotent state by promoting the transcription of *NANOG* close to an extent of in vivo produced blastocysts.

We didn’t find the reciprocal regulatory relationship [19] between improved expression of *IGF2* and unchanged expression of *H19* in case of raised DNA methylation levels after treatment by RG108 and scriptaid. Moreover, no obvious alterations of DNA methylation at the DMR2 region of *IGF2* were found. For the seemingly contradictory observations, we provided two possible explanations:Firstly,the shared enhancer elements that these two genes compete for [27] might be disrupted in cloned pre-implantation embryos;Secondly, other mechanisms independent of DNA methylation might exist because aberrant *IGF2* imprinting in human tumor cells could be repaired by unknown imprinting machinery in the normal fibroblast cytoplasm after nuclear transfer without any changes in DNA methylation [28]. In addition, in cloned mice, reduced expression of *H19* was also found not associated with increased expression of *IGF2* in case of hypermethylation of the *H19* DMR [29].

Imprinted genes have been proved susceptible for in vitro manipulations such as assisted reproductive technology in human [30], SCNT in animals [2], [15], [31], [32] and artificially induced reprogramming [33]. A previous report [15] and our study both observed disrupted imprinted methylation at *H19* locus during SCNT. Factors such as *DNMT1*, *DNMT3A*, *DNMT3L*, *ZFP57*, *MBD3*, were reported to exert important roles under specific circumstances [19], [34]–[36]. Encouragingly, we rescued the disrupted imprinted DNA methylation of ICR3 of *H19* in cloned embryos by addition of RG108 and scriptaid in culture medium for 17∼19 hours upon SCNT. Furthermore, we detected a significant reduced mRNA level of *MBD3*in RG+Scr-NT embryos at eight cell stage which was comparable to that in vitro fertilized counterpart, and further investigated that the rescued methylation levels at ICR3 of *H19* by RG108 and scriptaid could be reversed by overexpression of *MBD3* in cloned embryos. *MBD3* overexpression has been reported to induce DNA demethylation in an in vitro cellular model [37].Nevertheless, in normal mice embryos, *MBD3* was found essential for maintenance of methylation imprinting of *H19* in early mouse embryos [38]. Our results and these two findings would implicate that the balanced *MBD3* levels should be vital to proper DNA methylation reprogramming during early embryonic development.

We observed a dynamic process of de-methylation and re-methylation at the ICR3 region of *H19* in porcine cloned early embryos, which contradicted the knowledge that germ-line imprinted methylation could escape from DNA methylation reprogramming in early embryonic development [20], [21]. Our observation coincided with a previously report in porcine that ICR3 of *H19* undertook a dynamic reestablishment of imprinted methylation in early IVF embryos [39],but contrast to the results from another experiment in porcine where methylation of *H19* was maintained throughout pre-implantation development [15]. Considering the conflicting results around dynamic methylation adjustment and escape of globally methylation reprogramming of imprinted methylation in the early embryogenesis, we gave the brief explanation: (1) dynamic methylation adjustment do count as part of imprinting mechanisms [40]; (2) different methods adopted in artificial manipulation may complicate the experimental results.

We also fount RG108 and scriptaid could modify the reprogramming dynamics of *XIST* to the similar patterns as in IVF counterparts at the levels of both DNA methylation and transcription. DNA methylation has long been proved correlated with imprinted *XIST* expression and thus involved in regulating X chromosome inactivation [41], [42]. HDACi has also been reported to have inhibitory effect on *XIST* expression of human ESCs when adding sodium butyrate (one of HDACi) in culture medium [43]. These findings might provide explanations for our observations that scriptaid alone or along with RG108 modified the dynamics of *XIST* reprogramming during early embryonic development and repressed *XIST* expression at the blastocyst stage. Whether the inhibition of *XIST* in porcine early male cloned embryos would be beneficial to post-implantation development, might deserve further investigations, considering the phenomena occurred in mice that knockdown of *XIST* in male cloned pre-implantation embryos remarkably improved cloning efficiency [17].

The present study evaluated the effects of HDACi and DNMT1i on gene-specific transcription and DNA methylation in cloned embryos before implantation. The major conclusions included the following:(1) scriptaid alone and its combination with RG108 can improve developmental capacity and promote the transcription of *NANOG* before implantation; (2) combined treatment of constructed oocytes with scriptaid and RG108 can rescue the disrupted imprinted DNA methylation at ICR3 of *H19* by SCNT at least partially through repressing over-expressed MBD3 in cloned embryos at eight-cell stage; (3) RG108 and scriptaid can modify the reprogramming dynamics of *XIST* to a similar pattern in IVF counterparts which might contribute to porcine cloning efficiency.

## Materials and Methods

### Ethics Statement

This study was carried out in strict accordance with “The Instructive Notions with Respect to Caring for Laboratory Animals” issued by the Ministry of Science and Technology of China. The animal experimental protocol was approved by the Institutional Animal Care and Use Committee of South China Agricultural University. All efforts were made to minimize animal suffering.

### Ovary Collection and Oocyte Maturation

All batches of porcine ovaries used in this study were purchased from The Guangzhou Tianhe slaughterhouse located at Tianhe district, Guangzhou city, P. R. China. We obtained permission from this slaughterhouse to use the porcine ovaries for SCNT experiments in our study. Cumulus-oocyte complexes (COCs) were aspirated and matured in vitro for 42 ∼ 44 h following the protocol described by Deng et al [44]. Matured COCs were freed from cumulus cells by repeated pipetting in 0.1% hyaluronidase. Matured oocytes with the first polar body were selected for enucleation.

### Somatic Cell Nuclear Transfer

Preparation of cell lines and nuclear transfer manipulation were carried out as our previous description [45].

### In vitro Fertilization

Freshly ejaculated semen was obtained from a fertile and healthy Duroc boar, and after a short incubation at 39°C, the semen was mixed at an equal ratio and purified by a two-step Percoll gradient method. Briefly, semen was initially washed twice in Dulbecco’s Phosphate Buffered Saline (DPBS) supplemented with 0.1% (w/v) bovine serum albumin (BSA) by centrifugation at 250×g for 4 min. The pellet was resuspended and layered on the top of a 45∶90 discontinuous Percoli gradient and centrifuged at 300×g for 30 min. After discarding the final supernatant, the pellet was resuspended in 5 ml porcine gamate medium (PGM) medium [46] and washed twice at 150×g for 5 min. The spermatozoa were diluted with PGM to 2×10^6^ cells/ml and capacitated at 5% CO_2_ incubator at 39°C for 20 min. Matured oocytes were transferred into of PGM medium and incubated with capacitated spermatozoa for 6 hours; subsequently oocytes were washed three times.

### In vitro Cultivation Collection of Reconstructed Embryos

Activated reconstructed embryos and in vitro fertilized embryos were transferred into PZM3 medium and cultured at 39°C, 5% CO_2_, 7% O_2_, 88% N_2_ and 100% humidity. The time of embryo activation was defined as 0 h. Embryos at two-cell, eight-cell, morula and blastocyst stage were collected at 24 h, 72 h, 120 h and 168 h post-activation, respectively. The cleavage rate and blastocyst rate of cultured embryos were assessed at 24 h and 168 h. The total cell number of blastocysts was counted at day 168 h by staining the embryos with 1 µg/ml Hoechst33342 and viewing cell nuclei under a fluorescence microscopy.

### In vivo Derived Blastocysts

All the animal procedures were approved by the South China Agricultural University’s institutional animal care and use committee. Three Yorkshire sows were estrus detected and artificially inseminated with semen from the same Duroc boar over 2 days (Day 0 and 1). On Day six, sows were anesthetized with ketamine and xylazine for induction and 3% of isoflurane for maintenance. Embryos were flushed surgically with pre-warmed PBS containing 1% FCS and immediately transported to the lab in a portable incubator (Minitube).Embryos were isolated, graded and staged morphologically. We totally obtained twenty seven, thirteen and eleven morphologically normal blastocysts from three sows respectively. Subsequently, blastocysts were removed from zona pellucida by acid Tyrode solution at 37°C and then quickly transferred into 350 µL RLT Buffer provided with DNA/RNA Micro Kit (Qiagen) supplementing 10% 14.3 M β-mercaptoethanol. Vortex briefly and perform DNA and RNA extraction immediately or stored at −80°C up to one month. The embryos transferring manipulation was guaranteed to carry least solution.

### Preparation of RG108 and Scriptaid Stock Solutions

RG108 (10 mg; Sigma-Aldrich; R8279) was dissolved into 150 µL DMSO to make 200 mM stock solution (1000×). Scriptaid (1 mg; Sigma-Aldrich; S7817) were dissolved into 1.532 mL DMSO to get 2 mM stock solution and then transfer 5 µL into 95 µL DMSO to make 1000×SCR stock solution(100 µM).Divide into small tubes (5∼10 µL) and store at −80°C.

### Treatment of Constructed Embryos

When use, 1000×RG108 stock solution (RG-NT group), 1000×scriptaid stock solution (Scr100-NT group) were added to culture media. For “RG+Scr-NT group” treatment, 0.1% (v/v) 1000×RG108 stock solution and 0.1% (v/v) 1000×scriptaid stock solution were added simultaneously. After fusion, reconstructed oocytes were treated with drugs for 17∼19 hours according to experimental design.

### Embryos Recovery and Allocation

We allocated cloned embryos in each replicate according to the indexes to be observed, specifically, we made sure that five to ten blastocysts for counting total cells, at least thirty two-cell embryos, twenty eight-cell embryos, fifteen morulas or blastocysts for DNA and RNA extraction simultaneously, respectively. Each experiment included at least three replicates.

### Simultaneous Extraction of DNA and RNA from the Same Embryos Sample

All embryos samples kept in 350 µL Buffer RLT (Qiagen) were performed DNA and RNA extraction using an AllPrep DNA/RNA Micro Kit (Qiagen) followed kit recommendations. RNA was eluted with 14 µL RNase-free water and nearly 12 µL eluate was obtained. DNA was diluted in 40 µL pre-warmed elution buffer.

### Sexing of IVF Embryos

Individual morula or blastocyst was carried out DNA and RNA extraction and one third of the DNA was used to determine the sex and validate DNA extraction. The remaining DNA was subsequently pooled together for bisulfite conversion after sexing (Figure 9). Porcine *SRY* gene specific nested primers (Table S3 in File S2) were designed to distinguish the sex of IVF embryos. Nested PCR was run with the first round of 20cycles and the second round of 35 cycles. *ACTB* gene was used to verify the DNA extraction by single round of PCR with 45 cycles.

### Reverse Transcription and Relative Real-Time PCR

Before cDNA synthesis, genomic DNA was removed by incubation purified RNA with gDNA Wipeout Buffer provided with QuantiTect Reverse Transcription Kit (Qiagen) at 42°C for 2 minutes. Subsequently, the RNA samples were performed reverse transcription with the same kit according to manufacturer’s protocols. Real-time PCRs were carried out on an Illumina Eco (Illumina Inc, USA) using QuantiFast SYBR Green PCR Kit (Qiagen) in a 10 µL PCR reaction mix with 3 technical replicates. The thermal profile of all genes consisted of a denaturation cycles of 5 min at 95°C;45–50 cycles of amplification (95°C for 10 s,60°C for 30 s) and a melting cycle (95°C for 15 s, 55°C for 15 s, 95°C for 15 s). Relative expression levels of all analyzed genes were calculated relative to internal control gene (*ACTB*) and the reference sample (NT-con) by 2^−ΔΔCT^ method. All primers of analyzed gens were listed in Table S3 in File S2.

### Bisulfite Treatment of DNA ?

Purified genomic DNA from all replicates in each experiment were pooled and treated with sodium bisulfite to convert all unmethylated cytosine to uracil by using EZ DNA Methylation-Gold™ Kit (Zymo Research,Orange,CA) according to manufacturer’s recommendations. Briefly, 40 µL pooled DNA solution was denatured at 98°C for 10 min in 110 µL of CT Conversion Reagent (slight modification was made according to kit recommendations). Denatured DNA was incubated at 64°C for 2.5 h in the dark. Bisulfite-treated DNA was then desalted, purified, and diluted in 20 µL M-Elution Buffer. Subsequently, 4 µL converted DNA was used in the first run of the nested PCR amplification. For genes with a single round of PCR amplification, up to 10 µL could be used according to the copy number of the analyzed gene in genome.

### Bisulfite-specific PCR (BS-PCR) and Sequencing

Bisulfite modified DNAs were amplified with reported primers except *XIST* DMR2 which was designed with MethPrimer software. The primer sequences for DMR2 of *IGF2* and ICR3 of *H19* were described previously [19], the primers information for *XIST* were listed in Table 2. Nested PCRs were run using HotStarTaq plus DNA polymerase (Qiagen) with 30∼35 cycles of the first amplification reaction and 45 cycles of the second amplification reaction. The amplified products were verified by electrophoresis on 3% agarose gels and gel-purified using E.Z.N.A.® Gel Extraction Kit (Omega Bio-Tek). Purified PCR fragments were cloned into TA cloning vector pTZ57R/T (Fermentas). Positive colonies were confirmed by colony PCR and sent for sequencing until at least 14 qualified sequences at each DMR locus were obtained. Sequences were analyzed by local BiQ Analyzer software and bead-diagram was plotted on the web site (http://biq-analyzer.bioinf.mpi-inf.mpg.de/tools/MethylationDiagrams/index.php).

**Table 2:** Nested primers for bisulfite-specific PCR.

Gene	Primer sequences(5′ to 3′)		Annealing	Length(bp)	#CpG
***XIST-*** **DMR1**	Outer	F:TTATTTTGTAGATGGGAATTTATTG	50°C	490	11
		R:TACCTTAAAATATCCCAAATAACTA			
	Inner	F:TTTTTTTATTGGTTAAATTTTGAGT	55°C	190	6
		R:AAAAAATCCAATACCAACAAACTTC			
***XIST-*** **DMR2**	Outer	F:GTGTGTATTTTTTGATAAATTTTGT	55°C	330	16
		R:CRATACTAACTAACTAAATAAAAAC			
	Inner	F:GGATAATATGGTTGATTTTGTTATGTG	57°C	211	11
		R:CACCACCCTTTCTAATTAAATATATC			

### Porcine MBD3 Expression Plasmid

A Kozak sequence (in bold and italic in the forward primer) was added in front of the start codon and all restriction sites are underlined. The forward primer of NheI-Kozak-*MBD3* (5′-GCTAGC ***ACT***ATGGAGCGGAAGAGCCCAAG-3′) and reverse primer (5′-CTCGAGCTAGACGTGCTCCATCTCCTGGTC-3′) were used to amplify porcine *MBD3*. The 795 bp PCR product was purified and cloned into the vector pcDNA3.1+ (Invitrogen) using NheI/XhoI restriction sites. Plasmids were then sequenced and conformed.

### Statistical Analyses

Chi-square analysis was performed to compare the ratio of embryos at specific stage among different groups. For differential expression of all gens, 2^−ΔΔCT^ values were analyzed for normality and variance equality before arc-sine transformation. All data were presented as mean ± standard error of mean (SEM), and One-way ANOVA post hoc multiple comparisons (LSD method) analysis in SPSS 17.0 was used to compare differences among groups. Differences were considered significant at P<0.05.

## Supporting Information

File S1 Figure S1. The effects of concentrations of RG108 on donor adult fibroblast cells proliferation after 3 days in vitro culture. Cells were plated and conducted to mock treatment (A), treatment by 100 µM RG108 (B), treatment by 200 µM RG108 (C) and treatment by 100 µM RG108 (D). Figure S2. Identification of differentially methylated regions(DMRs) of porcine *XIST* gene 5′ flanking regions. Blasting X chromosome along sequence of EF619477.1 was conducted and an area containing two typical CpG islands were found (A-C). Transcription of two CpG islands (later defined as DMRs) (D-E) was carried out. Two CpG islands were differentially methylated in male and female genome of porcine adult fibroblasts (PFs) and therefore defined as DMRs (F).
(PDF)

File S2 Table S1. The effects of RG108 on development of porcine SCNT embryos in vitro. Effects of addition of three levels of RG108 (100 µM, 200 µM and 400 µM) in culture media upon SCNT on the developmental capacity (proportion of two-cell embryos, proportion of blastocysts and average total cells of blastocysts) were compared. Table S2. The effects of scriptaid on development of porcine SCNT embryos in vitro. Effects of addition of two levels of scriptaid (100 nM and 500 nM) in culture media upon SCNT on the developmental capacity (proportion of two-cell embryos, proportion of blastocysts and average total cells of blastocysts) were compared. Table S3. Primers for Real-Time RT-PCR or Embryo Sexing. Primers for fifteen genes concerned were listed.
(PDF)
