# Supplementary material for: Effects of DNMT1 and HDAC Inhibitors on Gene-Specific Methylation Reprogramming during Porcine Somatic Cell Nuclear Transfer
Source: PLoS One. 2013 May 31;8(5):e64705. doi: 10.1371/journal.pone.0064705 (PMC3669391; doi:10.1371/journal.pone.0064705)
Supplement: File S1 — Figure S1. The effects of concentrations of RG108 on donor adult fibroblast cells proliferation after 3 days in vitro culture. Cells were plated and conducted to mock treatment (A), treatment by 100 µM RG108 (B), treatment by 200 µM RG108 (C) and treatment by 100 µM RG108 (D). Figure S2. Identification of differentially methylated regions(DMRs) of porcine XIST gene 5′ flanking regions. Blasting X chromosome along sequence of EF619477.1 was conducted and an area containing two typical CpG islands were found (A-C). Transcription of two CpG islands (later defined as DMRs) (D-E) was carried out. Two CpG islands were differentially methylated in male and female genome of porcine adult fibroblasts (PFs) and therefore defined as DMRs (F). (PDF) [file pone.0064705.s001.pdf]

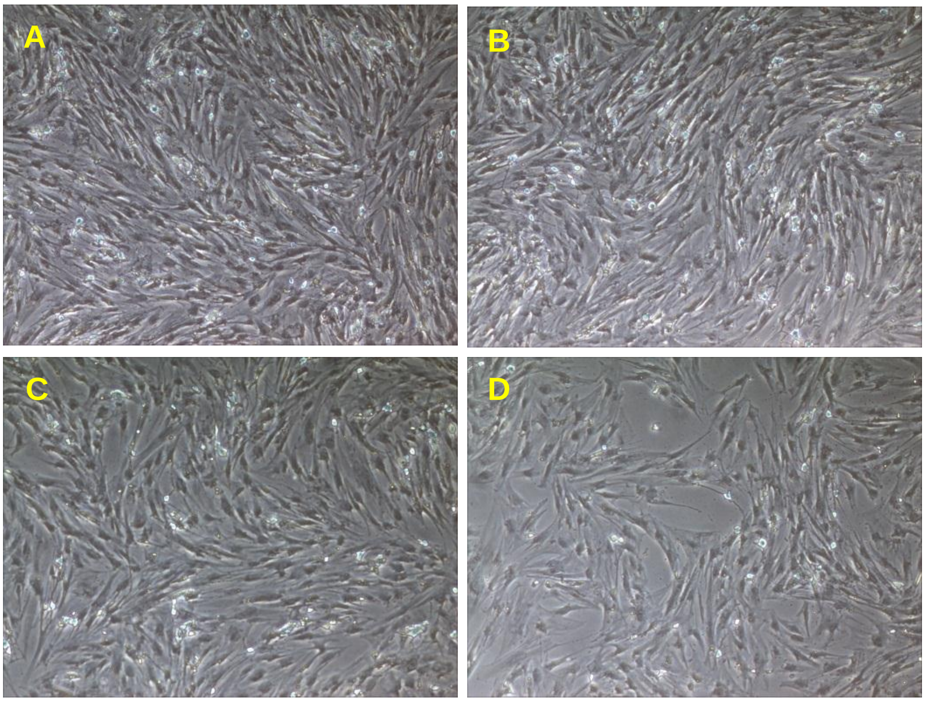

Figure S1. The effects of concentrations of RG108 on donor adult fibroblast cells proliferation after 3days in vitro culture. Cells were plated and conducted to mock treatment (A), treatment by 100 μM RG108 (B), treatment by 200 μM RG108 (C) and treatment by 400 μM RG108 (D).

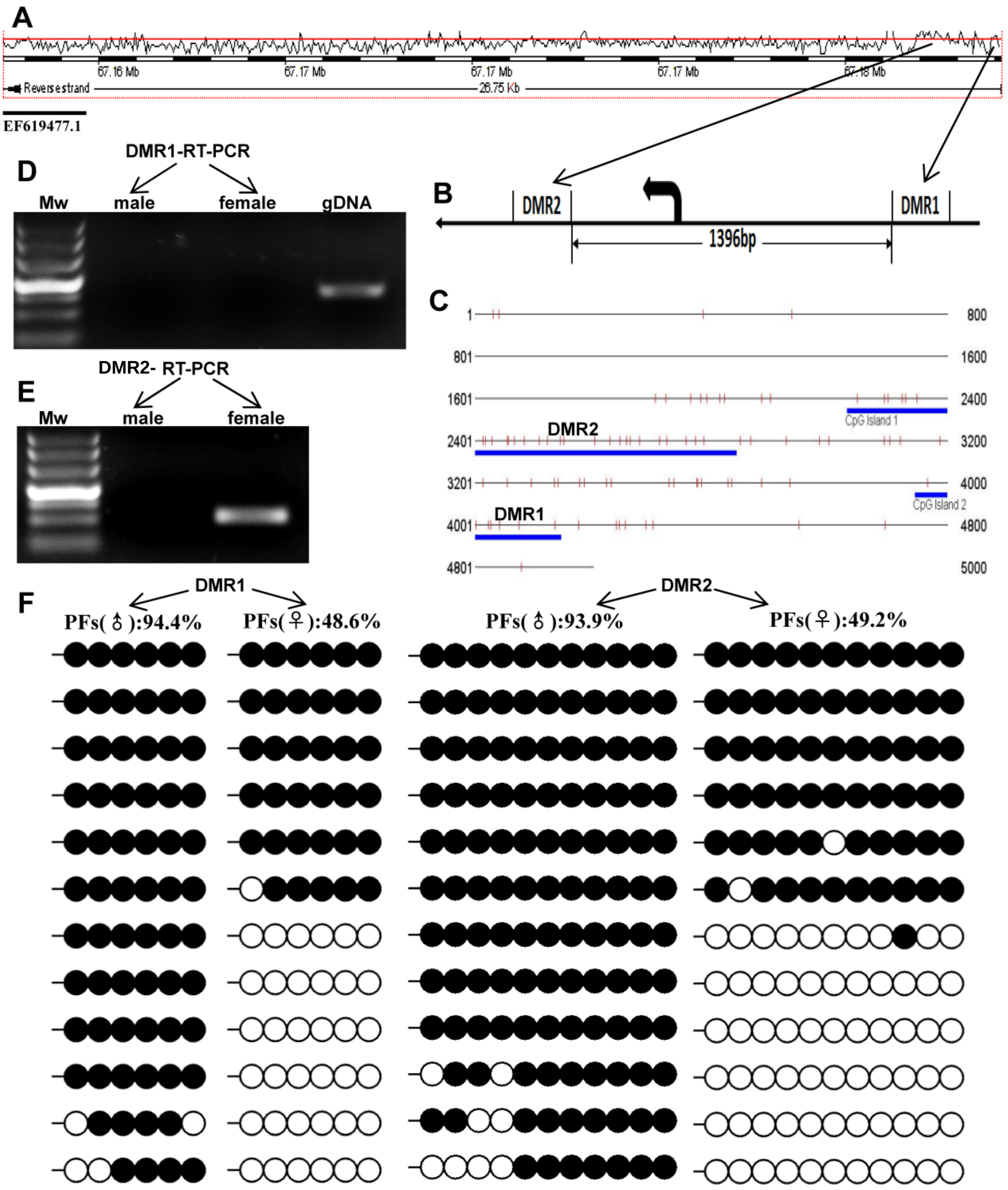

Figure S2. Identification of differentially methylated regions(DMRs) of porcine *XIST* gene 5′ flanking regions. (A-C) We blasted X chromosome along sequence of EF619477.1(partial mRNA sequence at 3′ UTR of *XIST*, reversely located at chrX: 58,373,888-58,374,926 in Sscrofa9.2) and found an area containing two typical CpG islands(reversely located at chrX: 58,400,130-58,400,319 , chrX: 58,398,546-58,398,756 in Sscrofa9.2,respectively) which were highly similar to promoter and exon1 regions of bovine (NR_001464.2) and horse (U50911.1).(D) DMR1 was not transcribed, while there had a transcribed region (E) which was located between DMR1 and DMR2, so we predicted transcription start site (TSS) of *XIST* might locate between two DMRs .The bent arrow (B) indicated the predicted TSS. (F)Two CpG islands were differentially methylated in male and female genome of porcine adult fibroblasts (PFs) and therefore defined as DMRs. We focused on DMR2 because it solely presented a dynamic methylation pattern.
