# Supplementary material for: Effects of DNMT1 and HDAC Inhibitors on Gene-Specific Methylation Reprogramming during Porcine Somatic Cell Nuclear Transfer
Source: PLoS One. 2013 May 31;8(5):e64705. doi: 10.1371/journal.pone.0064705 (PMC3669391; doi:10.1371/journal.pone.0064705)
Supplement: File S2 — Table S1. The effects of RG108 on development of porcine SCNT embryos in vitro. Effects of addition of three levels of RG108 (100 µM, 200 µM and 400 µM) in culture media upon SCNT on the developmental capacity (proportion of two-cell embryos, proportion of blastocysts and average total cells of blastocysts) were compared. Table S2. The effects of scriptaid on development of porcine SCNT embryos in vitro. Effects of addition of two levels of scriptaid (100 nM and 500 nM) in culture media upon SCNT on the developmental capacity (proportion of two-cell embryos, proportion of blastocysts and average total cells of blastocysts) were compared. Table S3. Primers for Real-Time RT-PCR or Embryo Sexing. Primers for fifteen genes concerned were listed. (PDF) [file pone.0064705.s002.pdf]

Table S1. The effects of RG108 on development of porcine SCNT embryos in vitro¶.

Treatment	% cleaved (n)	% blast (n)	Average total cells ± SEM
**Control**	76.67 (69/90)	24.44 (22/90)	40.00 ± 3.16 [ab]
**RG108-100μM**	74.55 (82/110)	23.64 (26/110)	35.29 ± 3.32 [a]
**RG108-200μM**	80.61 (79/98)	25.51 (25/98)	45.71 ± 2.89 [b]
**RG108-400μM**	69.09 (76/110)	25.45 (28/110)	35.20 ± 3.58 [a]

¶(1) the proportion of two-cell embryos (% cleaved) and the proportion of blastocysts (% blast) from 3 replicates; (2) n, number of embryos; (3) chi-square analysis was performed to compare the ratio of embryos; (4) one-way ANOVA post hoc multiple comparisons (LSD method) analysis in SPSS 17.0 was used to compare average total cells; (5) proportions or means labeled with the same letter or not labeled any letter do not differ from each other ($p > 0.05$) and without the same letter differ significantly ($p < 0.05$).

Table S2. The effects of scriptaid on development of porcine SCNT embryos in vitro.

Treatment	% cleaved (n)	% blast (n)	Average total cells ± SEM
**Con-NT**	70.13 (108/154)	10.39 (16/154) [a]	38.60 ± 2.37 [a]
**Scr500-NT**	71.03 (103/145)	20.00 (29/145) [b]	46.21 ± 3.49 [ab]
**Scr100-NT**	79.08 (121/153)	22.88 (35/153) [b]	52.43 ± 4.59 [b]

Table S3. Primers for Real-Time RT-PCR or Embryo Sexing.

Genes	Primer sequences(5′ to 3′)	Length(bp)	Resources
POU5F1	F: GAGAGGCAACCTGGAGAGCA	104	[1]
	R: CGCGGACCACATCCTTCTCT		
CDX2	F: AGAACCCCCAGGTCTCTGTCTT	198	
	R: CAGTCCGAAACACTCCCTCACA		
NANOG	F: AGGACAGCCCTGATTCTTCCACAA	101	[2]
	R: AAAGTTCTTGCATCTGCTGGAGGC		
DNMT1	F: GTGAGGACATGCAGCTTTCA	213	[3]
	R: AACTTGTTGTCCTCCGTTGG		
DNMT3A	F: CTGAGAAGCCCAAGGTCAAG	238	
	R: CAGCAGATGGTGCAGTAGGA		
HDAC2	F: TGGAGTACTGACTGCCTGGA	236	

	R: CCTGAACCTTTGTGGTGCTT		
IGF2	F: ACACCCTCCAGTTTGTCTGC	212	
	R: GGGGTATCTGGGGAAGTTGT		
BCL2	F: GAAACCCCTAGTGCCATCAA	196	
	R: GGGACGTCAGGTCACTGAAT		
BAX	F: AAGCGCATTGGAGATGAACT	251	
	R: CGATCTCGAAGGAAGTCCAG		
H19	F: GGCCGGAGAATGGGAAAGAAGG	148	[4]
	R: CGCAGTGCTGCGTGGGAACG		
KDM5C	F: CCTGGCAGCAGCTGTACATA	185	NM_001097433.1
	R: CATAGCAGGCTAGGGCTGAC		
MBD3	F: TGATTTCCGGACGGGCAAGATG	117	XM_003353980
	R: ACCGGGAGGGCTGTGTTCAAGTC		
XIST	F: CTTTGCCGCAGTGTTCCAGT	105	KC149530
	R: GCCGCCATCTTTTGCTAT		
ACTB	F: CCACGAGACCACCTTCAACTC	131	DQ845171
	R: TGATCTCCTTCTGCATCCTGT		
SRY	F: GCTTTCATTGTGTGGTCTCGT	309	NM_214452.3
**outer**	R: CTTGGCGACTGTGTATGTGAAG		
SRY	F: AGAACCCTCAAATGCAAAACTCA	115	
**inner**	R: ACCGCCTGTAGCCTCTGTG		

References

1 Ezashi T, Telugu BP, Alexenko AP, Sachdev S, Sinha S, et al. (2009) Derivation of induced pluripotent stem cells from pig somatic cells. Proc Natl Acad Sci U S A 106:10993-10998.

2 Bauer BK, Isom SC, Spate LD, Whitworth KM, Spollen WG, et al. (2010) Transcriptional profiling by deep sequencing identifies differences in mRNA transcript abundance in in vivo-derived versus in vitro-cultured porcine blastocyst stage embryos. Biol Reprod 83:791-798.

3 Kumar BM, Jin HF, Kim JG, Ock SA, Hong Y, et al. (2007) Differential gene expression patterns in porcine nuclear transfer embryos reconstructed with fetal fibroblasts and mesenchymal stem cells. Dev Dyn 236:435-446.

4 Wei Y, Zhu J, Huan Y, Liu Z, Yang C, et al. (2010) Aberrant expression and methylation status of putatively imprinted genes in placenta of cloned piglets. Cell Reprogram 12:213-322.
